# Supplementary material for: Direct structural observation of ultrafast photoisomerization dynamics in sinapate esters
Source: Commun Chem. 2022 Oct 31;5:141. doi: 10.1038/s42004-022-00757-6 (PMC9814104; doi:10.1038/s42004-022-00757-6)
Supplement: Supplementary file 1 — Supplementary Information [file 42004_2022_757_MOESM1_ESM.pdf]

Supplementary Information

Direct Structural Observation of Ultrafast Photoisomerization Dynamics in Sinapate Esters

Temitope T. Abiola[1], Josene M. Toldo[2*], Mariana T. do Casal[2], Amandine L. Flourat[3], Benjamin Rioux[3], Jack Woolley[1], Daniel Murdock[1], Florent Allais[3*], Mario Barbatti[2,4], Vasilios G. Stavros[1*]

1. Department of Chemistry, University of Warwick, Gibbet Hill Road, Coventry, CV4 7AL, United Kingdom.
   Email: v.stavros@warwick.ac.uk
2. Aix Marseille Université, CNRS, ICR, Marseille, France.
   Email : josene-maria.toldo@univ-amu.fr
3. URD Agro-Biotechnologies (ABI), CEBB, AgroParisTech, 51110, Pomacle, France.
   Email: florent.allais@agroparistech.fr
4. Institut Universitaire de France, 75231 Paris, France.

Supplementary Information Table of Content

Note S1: Additional FTIR and TVAS data 3
Note S2: Frequency calculations for assigning FTIR spectra 4
Note S3: Photoisomer determination 5
Note S4: Photoisomerization quantum yield determination 6
Note S5: Spectral decomposition with KOALA 8
Note S6: Transient electronic absorption spectra of ES 10
Note S7: Steady-state fluorescence measurements 12
Note S8: Geometries, normal modes, and vertical energies 13
Note S9: Multiconfigurational calculations 18
Note S10: Conical intersection characterization 21
Note S11: Linear interpolations in internal coordinates (LIICs) 23
Note S12: Topography of the conical intersections 24
Supplementary References 29

Note S1: Additional FTIR and TVAS data

The FTIR measurements of *E*-ES and *Z*-ES in acetonitrile and deuterated chloroform are shown in supplementary information Fig. S1. These data reveal that the FTIR spectra of both isomers are only mildly influenced by solvent polarity.

Supplementary information Fig. S2 shows the TVA spectra collected for *E*-ES and *Z*-ES, focusing on the region of the spectrum corresponding to the C=O band, in deuterated chloroform. These data reveal similar features to those obtained in acetonitrile and reported in the manuscript. As demonstrated in supplementary information Fig. S2, the positive band to the left of (i.e. lower frequency than) the ground state bleach (GSB, negative) feature decays to baseline on a similar delay time (Δt) of ~30 ps to that observed in acetonitrile. Together with the presence of the ESA at near Δt = 0, suggests that this feature is likely dominated by contributions from vibrational relaxation in the excited-state.

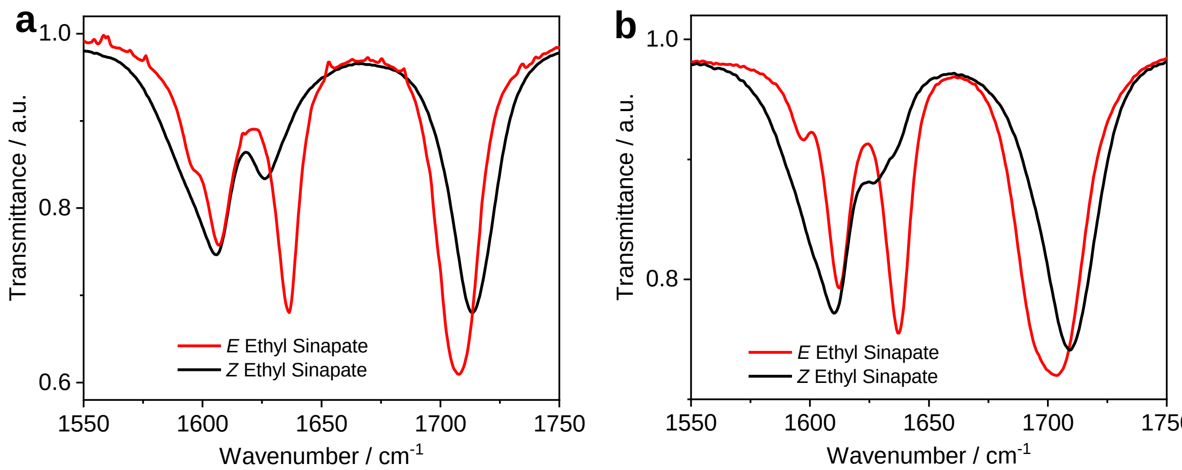

Fig. S1. Steady-state FTIR spectra of 20 mM solutions of *E*-ES and *Z*-ES in (**a**) acetonitrile and (**b**) deuterated chloroform over the 1550 cm^{-1} to 1750 cm^{-1} region.

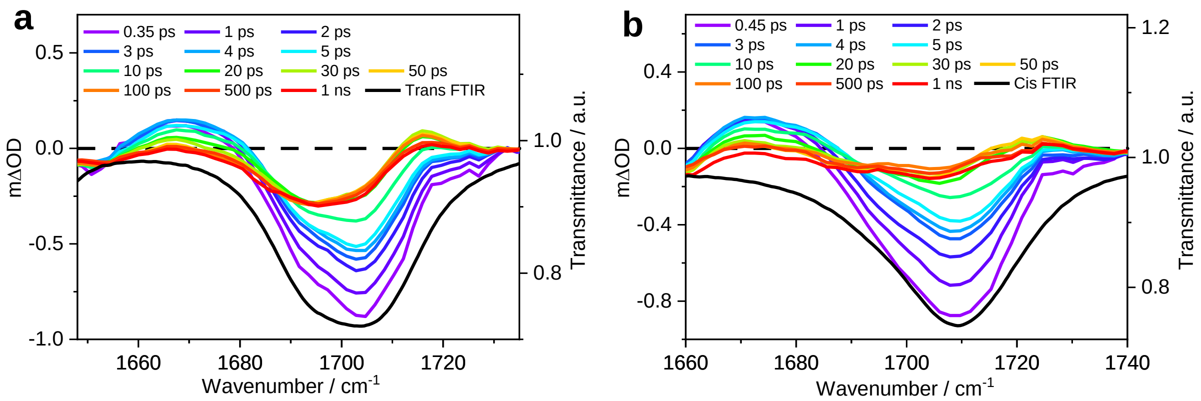

Fig. S2. TVA spectra of (**a**) *E*-ES and (**b**) *Z*-ES, both photoexcited with 320 nm in deuterated chloroform. The color key in panel (**a**) and (**b**) shows the time delays corresponding to the difference spectra. For both TVA spectra, the probe pulse is centered on ~1710 cm^{-1}.

Note S2: Frequency calculations for assigning FTIR spectra

Electronic ground-state geometry optimization was performed on different molecular conformers of *E*-ES and *Z*-ES with B3LYP/6-311++G** level of theory. The conformers with the lowest energy in each molecule were selected for further calculations. To assign vibrational modes to the experimentally observed FTIR bands, frequency calculations were carried out at the B3LYP/6-311++G** level of theory (PCM/acetonitrile) on the optimized S_0 geometry. The calculated S_0 frequencies and the associated vibrational modes are shown in supplementary information Table S1. For both predicted spectra (for *E*-ES and *Z*-ES), a scaling factor was calculated using one experimental peak as reference. This scaling factor was then applied to the calculated frequencies to match the reference experimental peak. This method has previously been employed for similar systems.[1-3] The selected reference peak for *E*-ES is 1708 cm^{-1}, and 1714 cm^{-1} for *Z*-ES, with resultant scaling factors of 0.9879 and 0.9908, respectively. As shown in Fig. 1 within the manuscript, the calculated strengths of these transitions have also been scaled to match the intensity of the selected reference peak. This confirmed that the calculated peak positions are in good agreement with the experimental bands.

Table S1. Computed S_0 vibrational frequencies (and transition intensities) for *E*-ES and *Z*-ES in acetonitrile, together with descriptions of the associated modes between 1550 and 1750 cm^{-1}, listed in order of increasing frequency. The structures of *E*-ES and *Z*-ES, with numbered atoms of interest, are also shown.

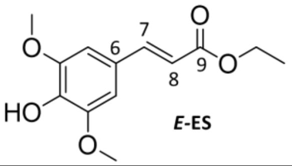		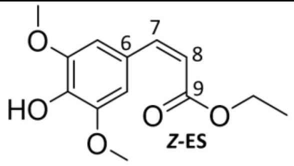	
wavenumbers /cm^{-1} (IR Int.)	Vibrational mode S_0	wavenumbers /cm^{-1} (IR Int.)	Vibrational mode S_0
1611 (38)	C-H in-plane bend (ar) + C=C stretch (ar)	1617 (38)	C-H in-plane bend (ar) + C=C stretch (ar)
1639 (35)	C_7=C_8 stretch	1634 (19)	C_7=C_8 stretch
1708 (51)	C_9=O stretch	1714 (40)	C_9=O stretch

(ar): aromatic

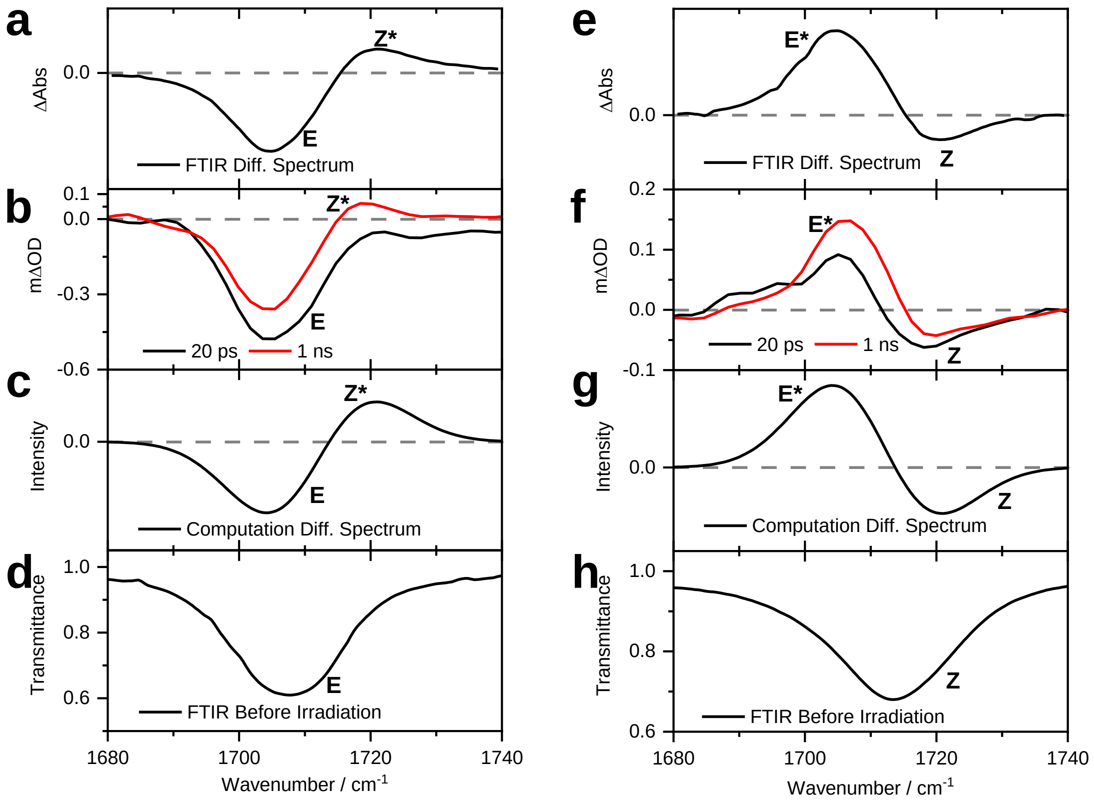

Fig. S3. Difference FTIR spectra of 20 mM acetonitrile solution of (**a**) *E*-ES and (**e**) *Z*-ES, between the spectrum after 6 hours of irradiation with a solar simulator and the spectrum before irradiation. TVA spectra of (**b**) *E*-ES and (**f**) *Z*-ES in acetonitrile at 20 ps and 1 ns pump-probe delay time. Difference spectra derived from the calculated C=O stretch frequency for (**c**) *E*-ES (bleach spectrum) and *Z*-ES (absorption spectrum), and (**g**) *Z*-ES (bleach spectrum) and *E*-ES (absorption spectrum). FTIR spectra of (**d**) *E*-ES and (**h**) *Z*-ES before irradiation.

Note S3: Photoisomer determination

We obtained both experimental and computationally predicted difference spectra for *E*-ES and *Z*-ES in order to confirm the assignment of the features centered at ~1717 cm^{-1} and ~1708 cm^{-1}, respectively, in acetonitrile transient vibrational absorption (TVA) spectra which start to appear at Δt of ~20 ps and persist until the longest Δt of 1 ns (see Fig. 2 in the main manuscript).

Supplementary information Fig. S3a and e show the steady-state difference IR spectra of *E*-ES and *Z*-ES in the region of the C=O stretch in acetonitrile. The difference spectra for both *E*-ES and *Z*-ES are obtained by subtracting the IR spectrum before irradiation from the IR spectrum acquired after 6 hours of irradiation with a solar simulator. In both difference spectra, the band below and above the zero line (horizontal, dashed) corresponds to the reactant and the photoproduct, respectively. In both spectra, the photoproduct frequency compares well with that of the corresponding isomer for each starting molecule. It is evident that the amplitude of this photoproduct band is lower than that of the reactant in *E*-ES (supplementary information Fig. S3a)

and higher than the reactant in *Z*-ES (supplementary information Fig. S3e). In both cases, this agrees with the TVA spectra at 1 ns (Fig. S3b and f) and confirms that the same photoproduct is generated in both steady-state and transient experimental data. Furthermore, the lower amplitude of *Z*-photoisomer formed from the starting *E*-isomer (and vice-versa) is in line with the steady-state FTIR spectra acquired before irradiation, which shows that the C=O stretch in *E*-ES has higher transmittance amplitude than that of *Z*-ES.

To further identify the origin of the photoproduct in both cases, we report the computational difference spectrum derived from the simulated vibrational spectra and compared it to those obtained from experiment. The simulated vibrational spectra were constructed from the calculated frequencies by convolution of the vibrational 'stick' spectra (see Fig. 1 in the main manuscript) with Gaussian functions with 20 cm^{-1} FWHM in the C=O stretch region. These computed Gaussian peaks were used either as ground-state bleach (negative signal) or excited-state absorption (positive signal) in supplementary information Fig. S3c and g. Linear addition of the computed C=O vibrational spectra for *E*-ES (as ground-state bleach band) and *Z*-ES (as excited-state absorption band) is shown in supplementary information Fig. 3c, and vice versa for Fig. 3g. The resulting data are in agreement with both steady-state difference spectra and TVA spectra at Δt = 1 ns, confirming that the positive feature observed in the 1 ns TVA spectra in both cases is the photoisomer.

Note S4: Photoisomerization quantum yield determination

In order to determine the photoisomerisation quantum yield (PQY) in both *E*-ES and *Z*-ES, the UV photostability measurements were recorded following single wavelength irradiation with an arc lamp as detailed in the methods within the manuscript. The plot of the changes in absorbance (at λ_{max}) of both isomers until photostationary state (PSS) is reached is shown in supplementary information Fig. S4. In line with prior expectations, for *E*-ES, the spectral profile reduced gradually until the PSS was achieved, while the spectral profile of *Z*-ES increased until the PSS was reached. This observation is due to the fact that, typically, *E*-isomers of sinapate esters have higher extinction coefficient than the corresponding *Z*-isomers (19 300 $M^{-1}cm^{-1}$ and 9 800 $M^{-1}cm^{-1}$ in the case of *E*-ES and *Z*-ES, respectively).[4] The PQY for both samples was quantitatively determined from the measurements reported above, using a series of equations previously reported by Hanson *et al.*,[5,6] briefly highlighted below with few modifications to the notation.

First, for each one of the starting isomers, we fit a mono-exponential function to the experimental changes in absorption ($A(\lambda)$) against time, as shown in supplementary information Fig. S4, given by:

$$A(\lambda, t) = A_{pss}(\lambda) + A_0(\lambda)e^{-kt} \quad (1)$$

where

$$k = k_{EZ} + k_{ZE} \quad (2)$$

k_{EZ} is the rate of conversion from *E*-ES to *Z*-ES, k_{ZE} is the rate of conversion from *Z*-ES to *E*-ES, and A_0 is the initial absorbance of the starting isomer. $A_{pss}(\lambda)$ is equivalent to the absorbance of the photostationary state and can be defined as:

$$A_{pss}(\lambda) = \frac{k_{EZ}}{k} N_0 \varepsilon_Z(\lambda) + \frac{k_{ZE}}{k} N_0 \varepsilon_E(\lambda) \quad (3)$$

Here, N_0 is the initial concentration of the starting isomer (20×10^{-6} M^{-1}) and ε_Z and ε_E are the extinction coefficients of *Z*- and *E*-isomers at the maximum absorbance.

Supplementary information Fig. S4a and b shows the changes in the absorption profile of *E*- and *Z*- isomer following irradiation; supplementary information Fig. S4c and S4d show the fitting of supplementary Equation (1) to their respective $\lambda_{max,}$ which allows us to extract both *k* and A_p for each of the starting isomers solutions. Obtaining A_p and *k* from the fit of equation 1, in turn, allows us to solve for k_{EZ} and k_{ZE} from supplementary Equations (2) and (3), which are associated to the reaction rates in the direct (reactant → product) and reverse (reactant ← product) directions. Assuming that photoisomerisation is the only process resulting in the formation of photoproducts, we then compute the photoisomerisation quantum yield ($\phi_{EZ}(\%)$ and $\phi_{ZE}(\%)$) for both starting isomers with supplementary Equations (4) and (5), resulting in the values given in supplementary information Table S2. To verify any solvent effect on the PQY, the same process discussed for data acquired in acetonitrile is repeated for the data obtained in deuterated chloroform, methanol and cyclohexane.

$$\phi_{EZ}(\%) = \frac{k_{EZ}}{k_{EZ} + k_{ZE}} \times 100\% \quad (4)$$

$$\phi_{ZE}(\%) = \frac{k_{ZE}}{k_{EZ} + k_{ZE}} \times 100\% \quad (5)$$

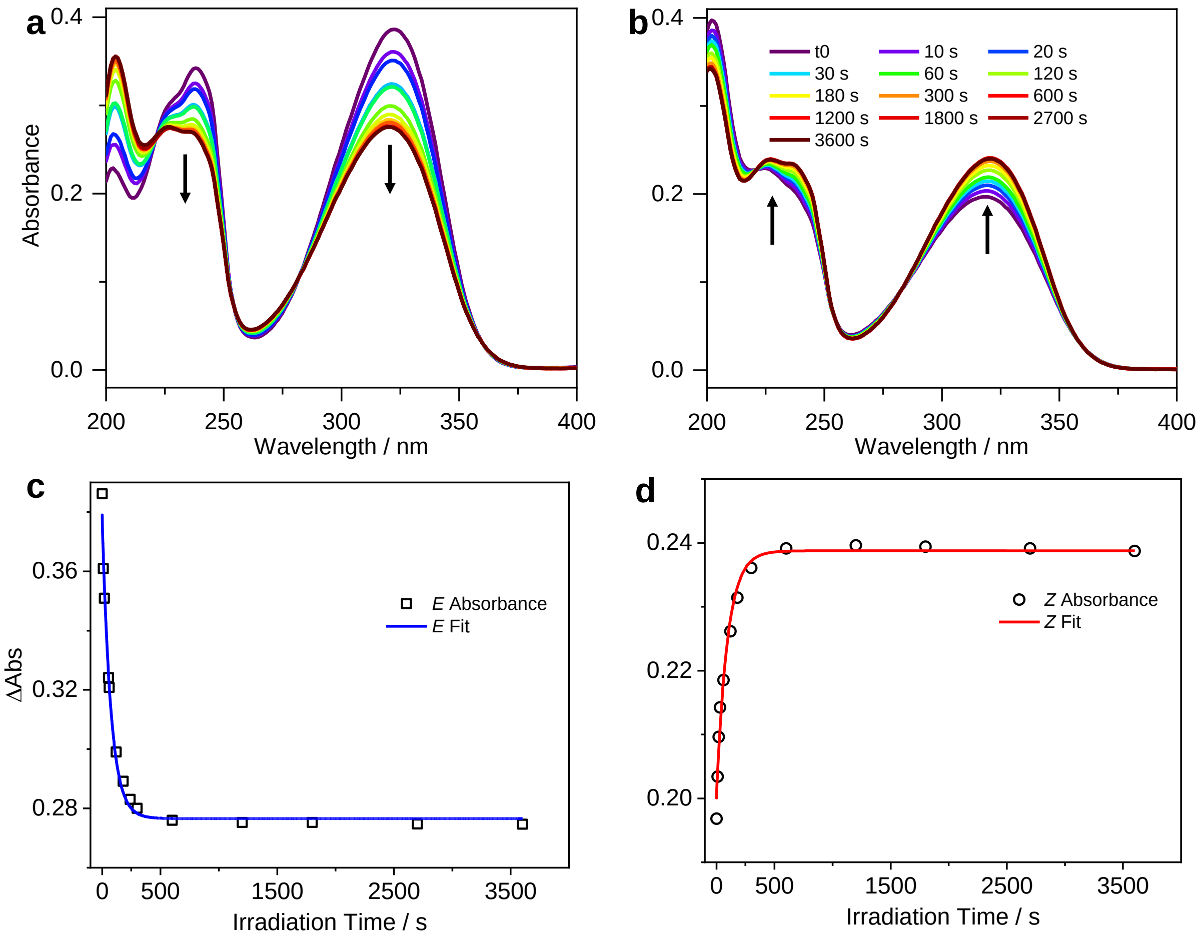

Fig. S4. UV-visible spectra obtained in acetonitrile for samples of (**a**) *E*-ES and (**b**) *Z*-ES at varying duration of irradiation with a xenon arc lamp. The downward and upward arrows denote the observed decrease and increase in absorbance over 60 minutes of irradiation, with the time color-coded in panel b. The mono-exponential fit to the change in absorbance over time until the photostationary state is reached is shown in (**c**) for *E*-ES and (**d**) *Z*-ES.

Table S2. PQY (ϕ_{EZ}(%) and ϕ_{ZE}(%)) calculated for the photoisomerisation reaction of either *E*-ES or *Z*-ES dissolved in acetonitrile, deuterated chloroform, methanol and cyclohexane following single wavelength irradiation with arc lamp

Starting Isomer	Acetonitrile		Deuterated chloroform		Cyclohexane	
	ϕ_{EZ} (%)	ϕ_{ZE} (%)	ϕ_{EZ} (%)	ϕ_{ZE} (%)	ϕ_{EZ} (%)	ϕ_{ZE} (%)
E-ES	57	43	56	44	56	44
Z-ES	77	23	86	14	71	29

Note S5: Spectral decomposition with KOALA

The decomposition of the TVA spectra of *E*-ES and *Z*-ES obtained in acetonitrile were achieved using three basis functions within the KOALA program.[7] The first function models the time-evolution of the ground-state bleach evident at ~1708 and ~1714 cm^{-1} in *E*-ES and *Z*-ES, respectively. The second function accounts for the decay of the vibrational relaxation, observed as a broad band at ~1690 cm^{-1} in both isomers. Finally, the third basis function is used to fit the

photoisomer feature that starts to grow in at approximately 10 ps. The absorption band resulting from the 10 ps growth is interpreted as representing the formation of the corresponding isomer resulting from photoisomerisation. Examples of TVA spectra decomposition at certain delay times are reported in supplementary information Fig. S5 for both *E*-ES and *Z*-ES in acetonitrile. The fit to the data is started at the instant of maximal GSB signal intensity, i.e., at a pump–probe delay of 0.9 ps in all cases, thereby avoiding any coherent artefacts at early time delays and effects attributable to solvent heating.[8]

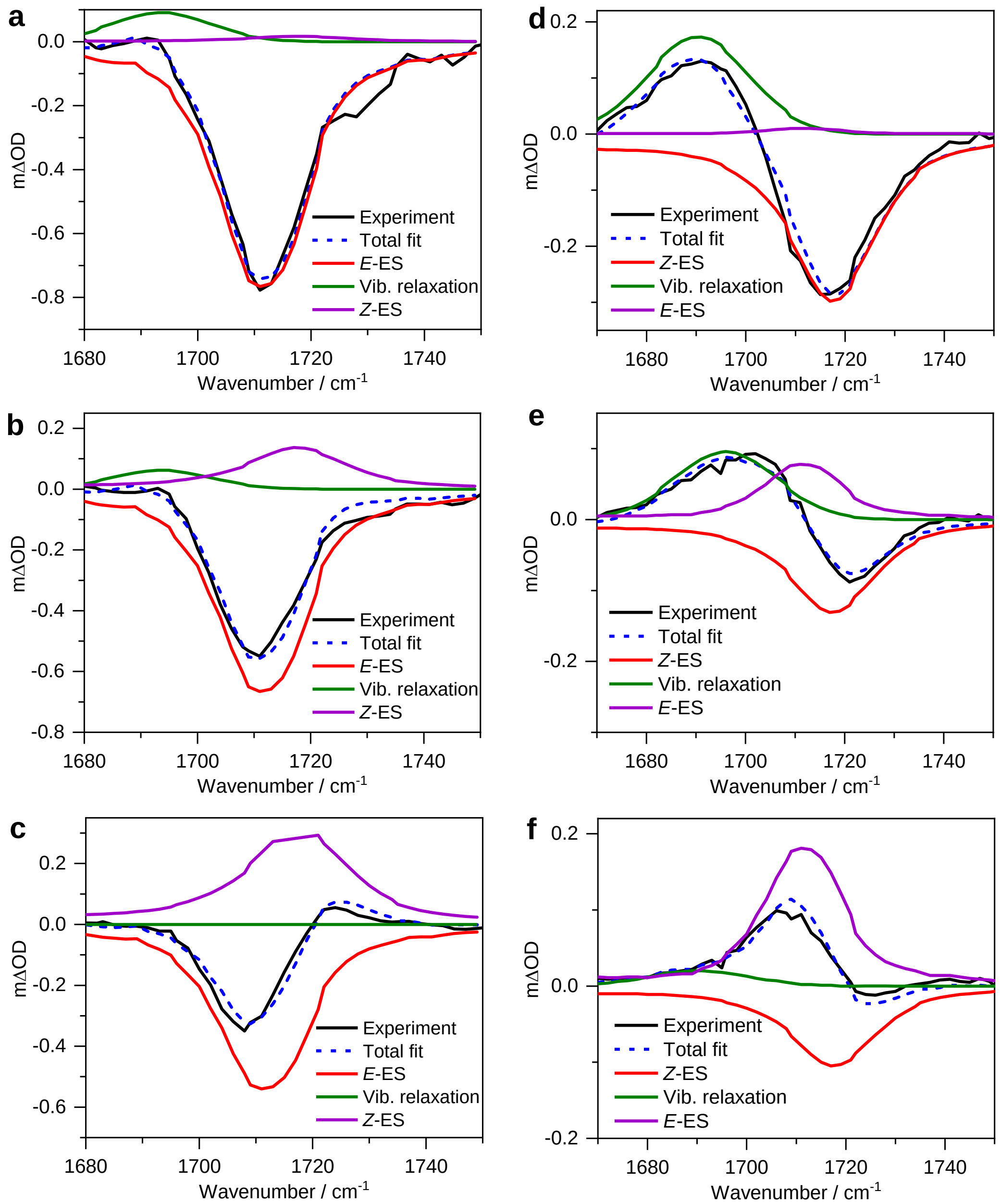

Fig. S5. Examples of decomposition of TVA spectra of *E*-ES in acetonitrile at delay times of (**a**) 1 ps, (**b**) 10 ps and (**c**) 80 ps. Decomposition for *Z*-ES is also shown at delay times of (**d**) 1 ps, (**e**) 10 ps and (**f**) 80 ps. The solid black line is the experimental spectrum, and the dotted blue line is the total fit from a combination of the three basis functions (shown in red, green, and purple). The red basis function is used to simulate the kinetics of the GSB. The green basis function tracks the kinetics of vibrational relaxation of photoisomers. Finally, the purple basis function accounts for the kinetics of photoisomer formation.

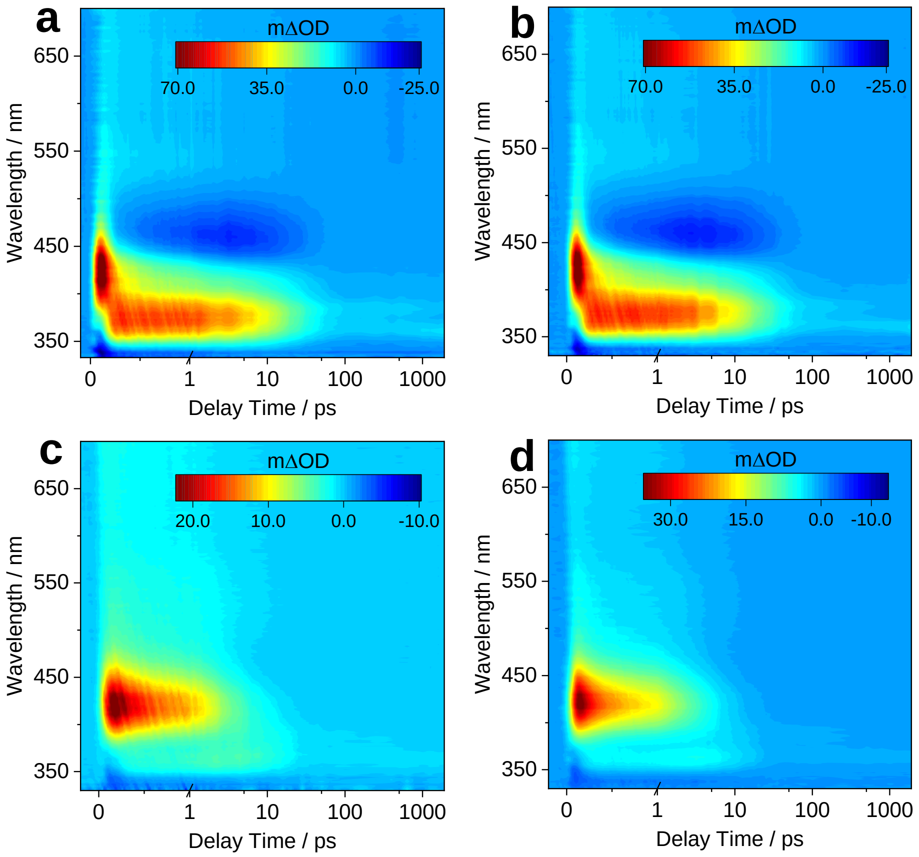

Fig. S6. TEA spectra obtained for 20 mM of (**a**) *E*-ES and (**b**) *Z*-ES in acetonitrile. Similar data are reported for (**c**) *E*-ES and (**d**) *Z*-ES in deuterated chloroform. Each molecule is photoexcited at their respective λ_{max} and the resulting spectra shown as false color maps. In each case, the pump-probe delay time is presented as a linear plot until 1 ps and then as a logarithmic scale between 1 and 100 ps.

Note S6: Transient electronic absorption spectra of ES

Previous TEAS measurements reported on sinapate esters, ES in particular,[4,9,10] have been carried out at a lower concentration (1 mM) compared to the concentration used for the TVAS measurements (20 mM) in the current study. Since the TVAS measurements give new insight into the kinetics of ES, it is reasonable to repeat the TEAS measurement at the same concentration used for the TVAS measurement. This measurement should reveal if a potential high concentration effect is responsible for the change in the kinetics of ES as revealed by the TVAS data. The TEAS measurements were carried out in both acetonitrile (polar solvent) and deuterated chloroform (non-polar solvent) and the resulting transient electronic absorption (TEA) spectra are reported in supplementary information Fig. S6. The data are very similar to previously reported spectra for ES (and other sinapate ester derivatives) in solvents of similar polarity at 1 mM concentration.[4,9] We briefly discuss the new insights into the TEAS data and the assigned dynamics herein.

The TEA spectra of *E*-ES and *Z*-ES in acetonitrile are dominated by four features, namely (*i*) ground state bleach (GSB) at ~340 nm (blue edge of the spectra), (*ii*) excited-state absorption (ESA) at ~420 nm, (*iii*) stimulated emission (SE) at ~460 nm, and (*iv*) a second broad ESA at the red end of the probe spectrum. In deuterated chloroform, all these features are observed except for the SE. The origin of the SE in acetonitrile and the possible cause for the absence in deuterated chloroform is explored below.

In acetonitrile, the ESA which is initially centered at ~ 420 nm is blue-shifted to ~380 nm with increased pump-probe delay time (Δt). This implies that as the systems evolves out of the Franck-Condon (FC) region, there are changes in $S_1 \rightarrow S_n$ transition character. We propose that this shift is a result of solute-solvent interaction which stabilizes the charge transfer state (*cf.* previous studies have demonstrated the charge character nature) of the molecule as it relaxes out of the FC region. This effect is not observed in deuterated chloroform due to the weak interaction between solute and solvent.

Furthermore, the observed SE in acetonitrile is a delayed process, i.e. not observed at $\Delta t = 0$, but begins to grow in at ~ 200 fs. This is likely due to the convolution of the ESA with the SE when the photoexcited molecules are in initially in the FC region. As the system relaxes out of the FC region, the SE feature begins to emerge. We note that the steady-state emission spectrum, shown in supplementary information Fig. S7, peaks at ~420 nm, lending support to the suggestion that the SE is initially convoluted with the strong ESA at ~420. However, as the system relaxes out of the FC region and the charge transfer state is stabilize by the polar solvent, the SE feature begins to appear. As a further note, since non-polar solvents will not stabilize charge transfer states (as is the case here), this could account for the absence of the SE in deuterated chloroform.

Finally, and as discussed in the main manuscript, the persistence of the SE feature (i.e. evident out to Δt ~50 ps) compared to the faster GSB recovery (*viz* ~10 ps) is likely an indication that some population must remain trapped in the excited-state for a longer time (S_1 minimum) and traverses through the CI on a longer timescale.

Kinetic information was extracted from the reported TEA spectra by applying a global sequential ($A \xrightarrow{\tau_1} B \xrightarrow{\tau_2} C \xrightarrow{\tau_3} D\ldots$) decay model, implemented through the Glotaran software package.[11,12] The extracted time constants are reported in supplementary information Table S3, and concur with previously reported values.[4,9] However, a new insight into the assignment of these time constant to dynamic processes is presented below.

The extracted time constants from the TEA spectra in both solvent environments are comparable to those reported for sinapate esters previously in solvents of similar polarity.[4,9] Based on the new

insight into the relaxation pathways from the TVAS experiment and the computational data reported in the main manuscript, we have now assigned a new dynamical process to the time constants. τ_1 and τ_2 are assigned to geometry evolution out of the FC region, and relaxation along the S_1 coordinate which then traverse through CI_E when starting with *E*-ES and CI_Z when starting with the Z-ES. Both of these processes are not observed in our TVAS measurements as we have excluded the early time delay data point (i.e. $\Delta t < 1$) from our TVAS fit to exclude the effects of solvent heating. The time constant associated with τ_3 (~19 ps in acetonitrile and ~13 in deuterated chloroform) is assigned to the time taken for the photoisomer formation. Previously, this time constant has been thought to include both the formation of the starting isomer and the photoisomer. That being said, the new insight provided by our TVAS data shows that a significant fraction of the starting isomer actually relaxes back to S_0 much faster. This new assignment proves that while TEAS is efficient in monitoring reaction pathways of photoactive materials, it may not be as effective in differentiating isomer specific timescales, as is the case for TVAS.

Table S3. Time constants and associated errors extracted from fitting the TEA spectra collected for *E*-ES and *Z*-ES in acetonitrile (top) and deuterated chloroform (bottom).

Solvent	Time constant	***E*-ES**	***Z*-ES**
Acetonitrile	τ_1 /fs	60 ± 40	60 ± 40
	τ_2 /fs	550 ± 40	560 ± 40
	τ_3 /ps	18.8 ± 0.2	18.7 ± 0.2
	τ_4 /ns	>2	>2
Deuterated Chloroform	τ_1 /fs	250 ± 40	160 ± 40
	τ_2 /ps	2.2 ± 0.1	1.9 ± 0.1
	τ_3 /ps	12.6 ± 0.2	11.1 ± 0.1
	τ_4 /ns	> 2	> 2

Note S7: Steady-state fluorescence measurements

The steady-state fluorescence measurements of both *E*-ES and *Z*-ES were obtained in both acetonitrile and deuterated chloroform. The resulting spectra are reported in supplementary information Fig. S7. Furthermore, the fluorescence quantum yield of *E*-ES (*Z*-ES) is determined to be 0.0053 (0.0063) and 0.0023(0.0035) in acetonitrile and deuterated chloroform, respectively.

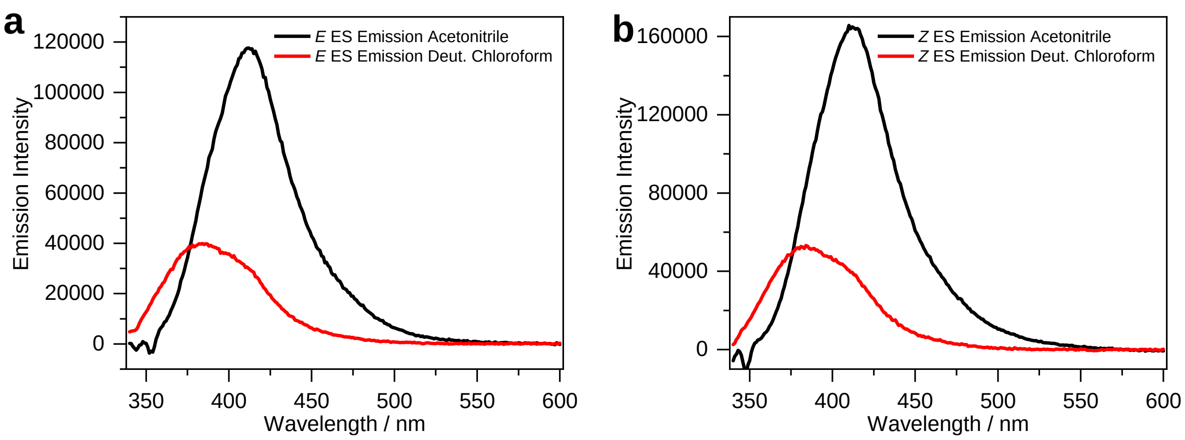

Fig. S7. Fluorescence emission spectra of (**a**) *E*-ES and (**b**) *Z*-ES. In both panels, the black line indicates the spectrum taken in acetonitrile while the red line indicates the spectrum taken in deuterated chloroform.

Note S8: Geometries, normal modes, and vertical energies

First, we present our initial investigation done at DFT and TDDFT level, where we compare the energies and frequencies for different conformers of *Z*-ES and *E*-ES.

The geometries of the ground and first excited singlet states were optimized using DFT and TDDFT calculations which employed CAM-B3LYP/cc-pVDZ in the gas phase.[13] Vibrational analysis was done at the same level of theory. Vertical energies were computed at the optimized geometries using CAM-B3LYP/aug-cc-pVDZ. All DFT/TDDFT calculations were done using Gaussian 16 Revision A.03.[14]

The optimized geometries, vertical energies, oscillator strengths, and selected geometrical parameters can be seen in supplementary information Fig. S8, Table S4, and S5, respectively. For each isomer, there are two conformers, *scis* and *strans*, as shown in supplementary information Fig. S8. The two conformers of the *E*-isomers differ by only 0.04 eV in the S_0, while the energy difference between the two conformers of the *Z*-isomers is more prominent (0.21 eV). All the conformers are planar in the ground-state except for *Z*-*strans*, which is slightly out-of-plane (as the dihedral angles of the C5-C6-C7-C8 and C6-C7-C8-C9 bonds are 171.3 and 2.8 degrees, respectively). The most stable conformer of the *Z*-isomer (*Z*-*scis*) is 0.12 eV less stable (i.e. higher in energy) than *E*-*scis* (the most stable *E* conformer).

The characterization of the excited-states at the ground-state geometry for the most stable conformers can be seen in supplementary information Fig. S9 and Fig. **S**10. The bright state (V state) has a $\pi\pi^*$ character and high oscillator strength; the S_2 is also a $\pi\pi^*$ state with smaller oscillator strength (V' state). The oscillator strengths computed for the S_1 state are larger for the *E*-isomer than for the *Z*-isomer, which agrees with the experimental UV-Vis absorption spectra.

The vertical excitations reasonably agree with the experimental absorption maximum reported in the main manuscript, i.e. 322 nm and 319 nm for *E*- and *Z*- isomers, respectively.

After photoexcitation to the S_1 state, both conformers of the *E*-isomer relax to a planar S_1 minimum while both conformers of the *Z*-isomers relax to a partially twisted geometry (~30° allylic torsion). The planar geometries for *Z*-isomers provided one imaginary frequency in the torsion direction. For *E*-isomers, the S_2 state is also planar and differs from the S_1 state by different bond length alternation (BLA). All attempts to optimize the S_2 state for the *Z*-isomers yielded the S_1-twisted geometry.

The inclusion of solvent effect (PCM/acetonitrile) does not change the order of the states, neither does it significantly shift the absorption wavelengths for the first excited-states (supplementary information Table S6).

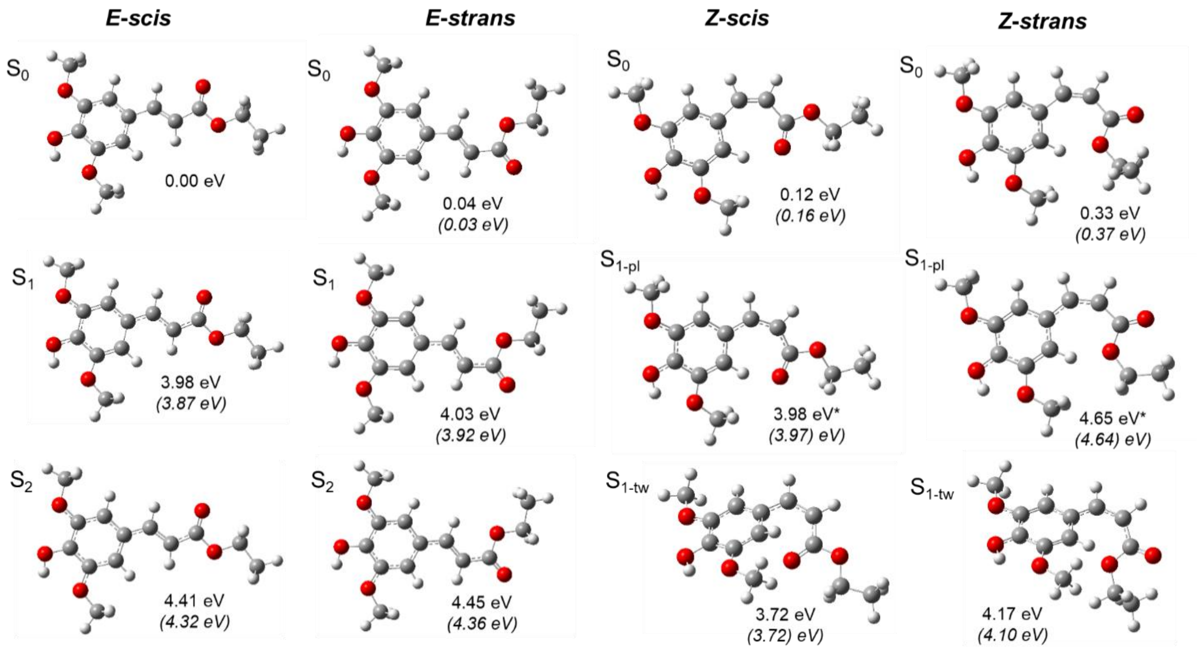

Fig. S8. Optimized structures for S_0, S_1 and S_2 states of *E*-ES and *Z*-ES isomers at CAM-B3LYP/cc-pVDZ level of theory. Relative energies (in relation to the most stable *E-scis* in the S_0 state) are given using cc-pVDZ and aug-cc-pVDZ (in parenthesis).

Table S4. *E*-ES conformers: absolute energies (in Hartree), vertical energies for S_0, S_1 and S_2 states and respective oscillator strengths (*f*), selected dihedral angles (in degrees) and bond lengths (in Å), and vibrational frequencies (not scaled, in cm^{-1}) computed for selected C=O and C=C stretching. Optimized structures at CAM-B3LYP/cc-pVDZ level. Vertical energies were calculated at the CAM-B3LYP/aug-cc-pVDZ //CAM-B3LYP/cc-pVDZ levels of theory.

	E-scis			***E-strans***		
	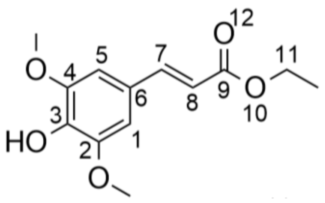			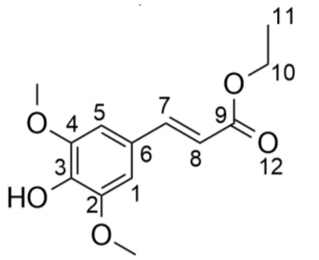		
	**S_0**	**S_1**	**S_2**	**S_0**	**S_1**	**S_2**
Energy (H) cc-pVDZ	-880.74774	-880.60133	-880.58565	-880.74623	-880.59951	-880.58409
Energy (H) aug-cc-pVDZ	-880.81547	-880.67333	-880.65663	-880.81425	-880.67132	-880.65513
S_1 vert (nm)	299	343	317	298	341	316
S_2 vert (nm)	273	287	302	273	287	302
S_3 vert (nm)	248	267	264	249	264	262
f (S_1)	0.625	0.635	0.651	0.608	0.620	0.629
f (S_2)	0.021	0.041	0.064	0.019	0.039	0.062
f (S_3)	0.000	0.000	0.000	0.000	0.000	0.000
Geometry						
C5-C6-C7-C8 (°)	180.0	180.0	180.0	180.0	-180.0	180.0
C6-C7-C8-C9 (°)	180.0	180.0	180.0	180.0	180.0	180.0
C7-C8-C9-O12 (°)	0.0	0.0	0.0	180.0	-180.0	-180.0
C6-C7 (Å)	1.461	1.417	1.425	1.463	1.419	1.427
C7-C8 (Å)	1.341	1.395	1.378	1.341	1.395	1.379
C8-C9 (Å)	1.474	1.442	1.444	1.473	1.443	1.443
C9-O12 (Å)	1.211	1.224	1.221	1.210	1.223	1.221
Frequencies						
ν C9-O12 (C=O) / (cm^{-1})	1826	1749	1765	1832	1775	1777
ν C7-C8 (C=C) / (cm^{-1})	1739	1695	1677	1735	1592	1666

Table S5. *Z*-ES conformers: absolute energies (in Hartree), vertical energies for S_0, S_1 and S_2 states and respective oscillator strengths (*f*), selected dihedral angles (in degrees) and bond lengths (in Å), and vibrational frequencies (in cm^{-1}) computed for selected C=O and C=C stretching. Optimized structures at CAM-B3LYP/cc-pVDZ level. Vertical energies were calculated at the CAM-B3LYP/aug-cc-pVDZ //CAM-B3LYP/cc-pVDZ levels of theory. Note that S_1 planar is not a minimum since it has one imaginary frequency corresponding to the twisting mode.

	Z-scis			***Z-strans***		
	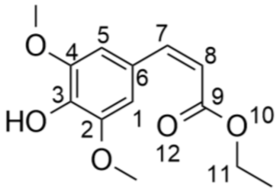			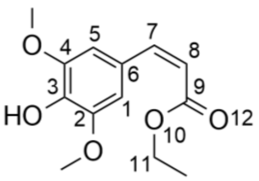		
	**S_0**	**$S_{1\ twisted}$**	**$S_{1\ planar}$** (i 31 cm^{-1})	**S_0**	**$S_{1\ twisted}$**	**$S_{1\ planar}$** (i 148 cm^{-1})
Energy (H) cc-pVDZ	-880.74332	-880.61091	-880.60150	-880.73559	-880.59462	-880.57684
Energy (H) aug-cc-pVDZ	-880.80968	-880.67888	-880.66973	-880.80187	-880.66498	-880.6451
S_1 vert (nm)	306	588	346	299	385	335
S_2 vert (nm)	283	347	300	277	308	290
S_3 vert (nm)	254	279	272	248	277	270
f (S_1)	0.529	0.033	0.515	0.499	0.348	0.531
f (S_2)	0.041	0.213	0.072	0.035	0.061	0.066
f (S_3)	0.000	0.029	0.000	0.000	0.009	0.000
Geometry						
C5-C6-C7-C8 (°)	180.0	-150.3	-180.0	171.3	-171.7	180.0
C6-C7-C8-C9 (°)	0.0	13.7	0.0	-2.8	36.628	0.000
C7-C8-C9-O12 (°)	0.0	1.4	0.0	179.6	-171.3	-180.0
C6-C7 (Å)	1.462	1.435	1.431	1.467	1.422	1.428
C7-C8 (Å)	1.351	1.394	1.401	1.350	1.405	1.403
C8-C9 (Å)	1.472	1.404	1.442	1.476	1.442	1.450
C9-O12 (Å)	1.215	1.280	1.237	1.211	1.226	1.224
Frequencies						
ν C9-O12 (C=O) / (cm^{-1})	1803	1489	1728	1813	1753	1751
ν C7-C8 (C=C) / (cm^{-1})	1721	1591	1589	1720	1552	1583

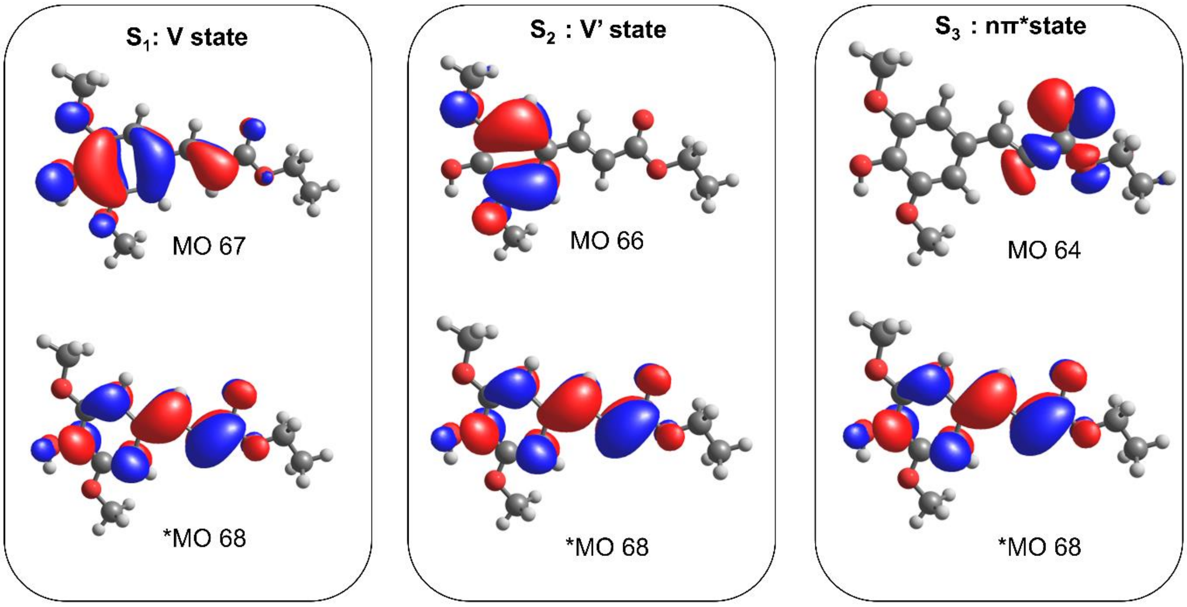

Fig. S9. Characterization of the vertical transitions for *E-scis*. Vertical transitions from the ground-state optimized at CAM-B3LYP/cc-pVDZ level of theory. These transitions are analogous at CASSCF level.

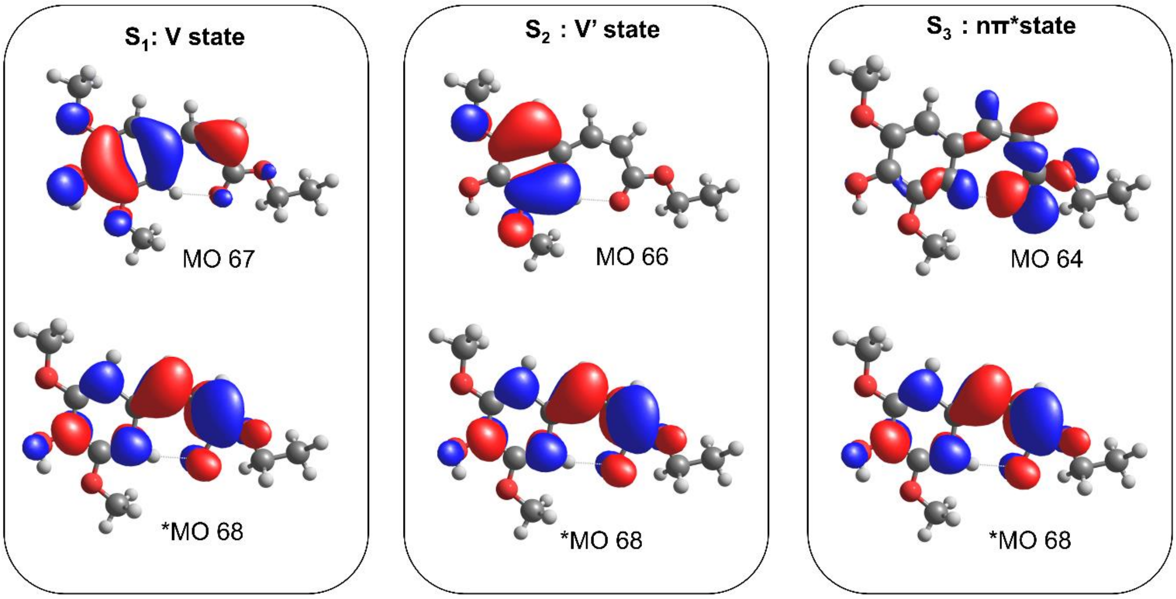

Fig. S10. Characterization of the vertical transitions for *Z-scis*. Vertical transitions from the ground-state optimized at CAM-B3LYP/cc-pVDZ level of theory. These transitions are comparable to those obtained with calculations with CASSCF.

Table S6. Vertical energies for the conformers of the *Z*-ES and *E*-ES isomers computed in acetonitrile (ACN) and respective oscillator strengths. Optimized structures at CAM-B3LYP/cc-pVDZ level. Vertical energies were calculated at the CAM-B3LYP/aug-cc-pVDZ //CAM-B3LYP/cc-pVDZ levels of theory. Their S_0 relative energies, ΔE, (in EV) is also given.

**ACN/ PCM**	***E-scis***		***E-strans***		***Z-scis***		***Z-strans***	
	**ΔE (eV)**	**f. osc.**	**ΔE(eV)**	**f. osc.**	**ΔE(eV)**	**f. osc.**	**ΔE(eV)**	**f. osc.**
S_1	308	0.7510	307	0.7371	309	0.6435	306	0.5594
S_2	279	0.0298	279	0.0288	284	0.0348	281	0.0332
S_3	242	0.0001	241	0.0000	252	0.0000	242	0.0009
ΔE S_0 (eV)	0.00		0.02		0.24		0.37	

Note S9: Multiconfigurational calculations

To investigate the photodeactivation pathways, we calculated the S_1 potential energy surface for the most stable conformer of each isomer, *Z-scis* and *E-scis* at the XMS-CASPT2/CASSCF level of theory. From now on, these isomers will be referred simply as *Z*-ES and *E*-ES, respectively.

Starting from the previously optimized structures at the CAM-B3LYP functional, state-average complete active space self-consistent field (SA-CASSCF)[15] was used to optimize S_0, S_1, S_2, and conical intersections in the gas phase. The thus calculated energies were subsequently corrected using multi-state complete active space second-order perturbation (MS-CASPT2)[16] and extended MS-CASPT2 (XMS-CASPT2)[17]. In all cases, a 6-31G(d) basis set was used. For all calculations, an IPEA[18] shift of 0.25 a.u. and an imaginary shift[19] of 0.1 a.u. were used. All CASPT2//CASSCF calculations were done using OpenMolcas v.19.11 (tag 283-ge7efbbb).[20]

Two different active spaces (AE) were chosen. The first one comprises of 6 electrons in 6 orbitals, here referred to as “small AE” (supplementary information Fig. 11) and weighted over 4 states for S_0, S_1 and S_2 optimizations and over 2 states for the conical intersections (CIs). In this case, only 3π and 3π* were included in the active space. The second active space is larger and comprising 12 electrons in 11 orbitals, here referred to as “large AE” (supplementary information Fig. S12), and equally weighted over 5 states. This active space includes 5π, 5π* and one lone pair of the carbonyl oxygen. Geometry optimizations were done using the small active space, while the energies were computed using the large one. The optimization of S_0, S_1, and S_2 found a planar minimum for these structures for both isomers. Optimization of these states using the large and small active spaces resulted in similar geometries as shown in supplementary information Fig. S13 and Table S7. For these geometries, a strong mixing between V and V’ states (S_1 and S_2 states at XMS-CASPT2 level) was observed, similar to what was reported in ‘structure-related’ molecules.[21] Similarly, this mixing can be explained due to an excitonic-like coupling of the

ethylenic ππ* state and the aromatic L_b state. A similar S_1 and S_2 mixing was found using either large or small active spaces. It is worth noticing that the reference weights did not improve by increasing the AE from (6,6) to (12,11). Vertical excitation energies are given in supplementary information Table S8. Moreover, we notice a significant effect of the dynamical correlation: the V state is the fifth root in the SA5-CASSCF calculations but drops to root two (i.e. S_1 state) at MS- or XMS-CASPT2 (supplementary information Table S9). Due to states-mixing, we have chosen XMS-CASPT2 for the subsequent analysis. Therefore, along the text we refer to the V and V' states as S_1 and S_2, respectively. Relaxation of S_1 and S_2 states out of the Franck-Condon region occurs along the bond length alternation (BLA) coordinate yielding planar structures with different double and single bond length orders (i.e. different BLA). The S_1 planar minimum found by SA-CASSCF optimization is consistent with TDDFT calculations for the *E*-isomer. The *Z*-ES presents a planar structure at CASSCF calculations but a slightly twisted geometry at TDDFT level.

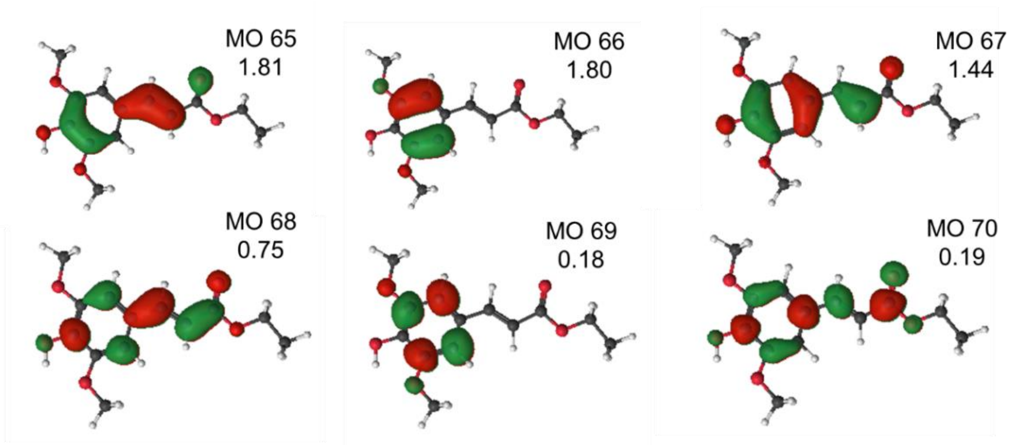

Fig. S11. Orbitals selected for the active space in SA4-CASSCF(6,6) calculations. Occupation numbers of the natural orbitals are given in parenthesis.

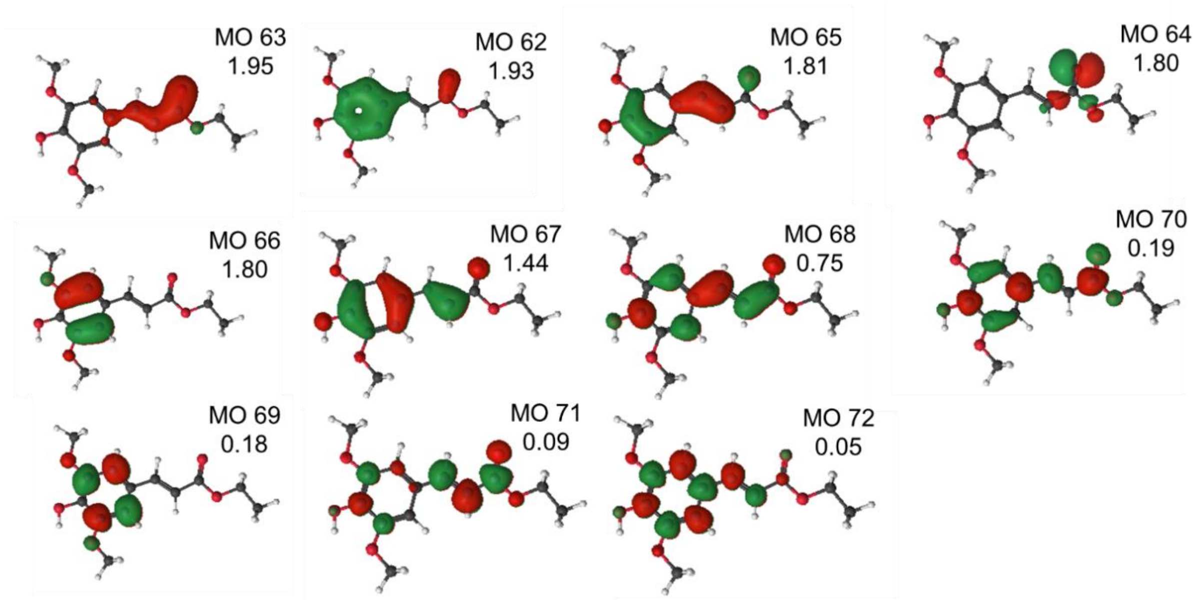

Fig. S12. Orbitals selected for the active space in SA5-CASSCF(12,11) calculations. Occupation number of the natural orbitals are given in parenthesis.

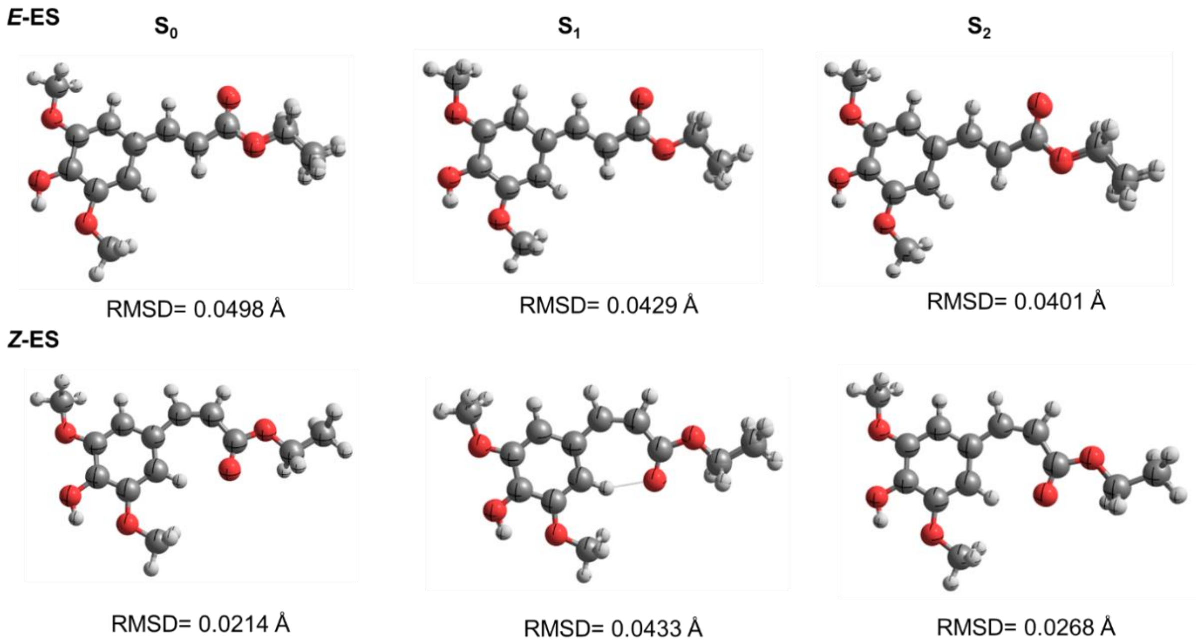

Fig. S13. Comparison between the geometries optimized with large and small active spaces: superposed minimum energy structures at SA4-CASSCF(6,6) and SA5-CASSCF(12,11) and respective RMSD.

Table S7. Selected geometrical parameters for *E*-ES and *Z*-ES optimized at SA4-CASSCF(6,6) and SA5-CASSCF(12,11). Atom numbering is shown in Table S4.

	**CASSCF(6,6)**			**CASSCF(12,11)**		
E-ES	**S_0**	**V state**	**V' state**	**S_0**	**V state**	**V' state**
C5-C6-C7-C8 (°)	-180.0	-180.0	-180.0	-180.0	180.0	180.0
C6-C7-C8-C9 (°)	180.0	-180.0	-180.0	180.0	-180.0	-180.0
C7-C8-C9-O12 (°)	0.0	0.0	0.0	0.0	0.0	0.0
C6-C7 (Å)	1.485	1.404	1.441	1.469	1.391	1.418
C7-C8 (Å)	1.315	1.383	1.333	1.348	1.428	1.374
C8-C9 (Å)	1.487	1.446	1.475	1.480	1.414	1.471
C9-O12 (Å)	1.191	1.203	1.194	1.197	1.225	1.199
Z-ES						
C5-C6-C7-C8 (°)	180.0	179.7	179.9	-180.0	180.0	-180.0
C6-C7-C8-C9 (°)	0.0	0.5	0.1	0.0	0.1	0.0
C7-C8-C9-O12 (°)	0.0	-0.3	0.0	-0.1	1.4	0.0
C6-C7 (Å)	1.486	1.418	1.432	1.471	1.392	1.418
C7-C8 (Å)	1.322	1.398	1.350	1.356	1.447	1.385
C8-C9 (Å)	1.489	1.437	1.468	1.480	1.412	1.469
C9-O12 (Å)	1.191	1.214	1.199	1.199	1.231	1.202

Table S8. Vertical excitation energies from the ground-state geometry computed at different multiconfigurational levels for *E-scis* and *Z-scis* isomer.

	MS-CASPT2(12,11) //SA-CASSCF(6,6)		XMS-CASPT2(12,11) //SA-CASSCF(6,6)		MS-CASPT2(12,11) //SA-CASSCF(12,11)		XMS-CASPT2(12,11) //SA-CASSCF(12,11)	
E-ES	*Exc. Energy*	*f*	*Exc. Energy*	*f*	*Exc. Energy*	*f*	*Exc. Energy*	*f*
S_1 (ππ*)	4.51	0.578	4.91	0.544	4.43	0.615	4.81	0.632
S_2 (ππ*)	4.72	0.012	4.98	0.064	4.68	0.007	4.95	0.009
S_3 (nπ*)	5.45	0.001	5.37	0.001	5.26	0.001	5.18	0.001
Z-ES	*Exc. Energy*	*f*	*Exc. Energy*	*f*	*Exc. Energy*	*f*	*Exc. Energy*	*f*
S_1	4.37	0.546	4.80	0.554	4.28	0.568	4.70	0.568
S_2	4.69	0.001	4.96	0.002	4.66	0.003	4.92	0.003
S_3 (nπ*)	5.39	0.001	5.32	0.001	5.22	0.001	5.14	0.001

Table S9. Absolute energies (in Hartree), adiabatic energies (ΔE, in eV), and dominant state configurations for S_0, S_1 and S_2 states optimized at SA4-CASSCF(6,6) and SA5-CASSCF(12,11).

	SA4-CASSCF(6,6)			SA5-CASSCF(12,11)		
E-ES	**S_0**	**V state**	**V' state**	**S_0**	**V state**	**V' state**
Configuration	222000	22ud00	2u2d00	22222200000	22222ud0000	2222u2d0000
Energy (H) CASSCF	-875.94038	-875.72797	-875.77464	-875.99837	-875.78498	-875.83173
Energy (H) MS-CASPT2	-878.40978	-878.26574	-878.25047	-878.40791	-878.24610	-878.24365
Energy (H) XMS-CASPT2	-878.41886	-878.25660	-878.25005	-878.41745	-878.25157	-878.24337
ΔE (eV) SA-CASSCF	0.00	5.78	4.51	0.00	5.81	4.53
ΔE (eV) MS-CASPT2	0.00	3.92	4.33	0.00	4.40	4.47
ΔE (eV) XMS-CASPT2	0.00	4.42	4.59	0.00	4.51	4.74
Z-ES	**S_0**	**V state**	**V' state**	**S_0**	**S_1**	**S_2**
Configuration	222000	22ud00	2u2d00	22222200000	22222ud0000	2222u2d0000
Energy (H) CASSCF	-875.93120	-875.72331	-875.76756	-875.98973	-875.78365	-875.82379
Energy (H) MS-CASPT2	-878.40346	-878.26654	-878.24811	-878.40254	-878.24661	-878.23917
Energy (H) XMS-CASPT2	-878.41277	-878.25770	-878.24962	-878.41203	-878.25319	-878.23849
ΔE (eV) SA-CASSCF	0.00	5.66	4.45	0.00	5.61	4.52
ΔE (eV) MS-CASPT2	0.00	3.73	4.23	0.00	4.24	4.45
ΔE (eV) XMS-CASPT2	0.00	4.22	4.44	0.00	4.32	4.72

Note S10: Conical intersection characterization

The search for conical intersections (CIs) was performed using SA-CASSCF(6,6)/6-31G(d) averaged over two states. The energies were subsequently corrected by single-point calculations using XMS-CASPT2(12,11) averaged over five electronic states.

The structures for the different S_1/S_0 CIs identified are shown in supplementary information Fig. S14. Selected geometrical parameters of these CIs can be seen in supplementary information Table S10 and their relative energies in Table S11. The most marked difference between them is the torsional angle ϕ (C6-C7-C8-C9) followed by the H-C7-C8-H torsional angle (HOOP). The most stable conical intersection, CI_{PYR}, has a torsional angle ~80° twisted from the original isomer and presents pyramidalization in the C8 atom. CI_{PYR*} is equivalent to CI_{PYR} but twisted in the opposite direction. Highly twisted geometries without this pyramidalization exhibit higher energies, i.e. CI_{Perp}. Two other S_1/S_0 conical intersections (CI_E and CI_Z) were located with energies slightly above CI_{PYR}. They have smaller torsional angle, but only CI_E presents pyramidalization in the C8 atom. These intersections are likely connected to CI_{PYR} through an intersection seam along the twisting coordinate. An ethylidene ($CI_{ethylidene}$) and a hula-twist (CI_{HT}) CI were also located but with higher energies. Therefore, we focused our attention on the characterization of the branching planes involving only the most stable CI_E, CI_Z and $CI_{PYR}/_{PYR*}$.

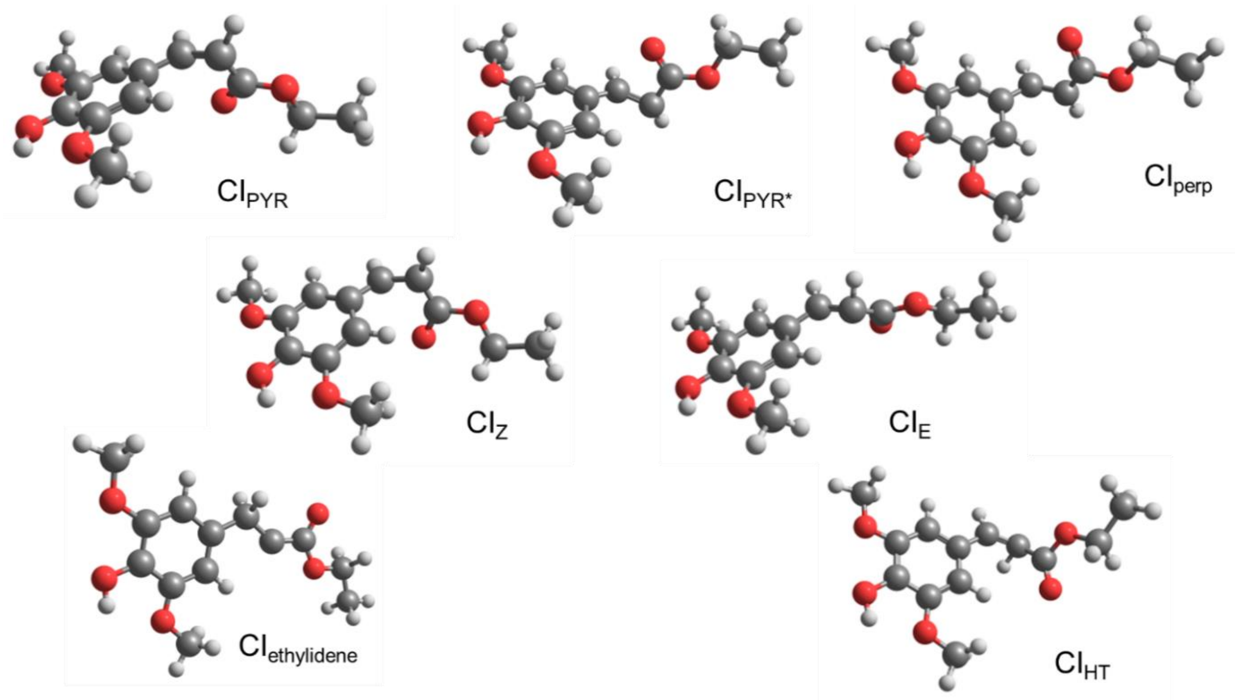

Fig. S14. Structures of the conical intersections optimized at SA2-CASSCF(6,6) level.

Table S10. Selected geometrical parameters for conical intersections optimized at SA2-CASSCF(6,6) level. Dihedral angles and angles in degrees and bond lengths in Å.

CI	C5-C6-C7-C8	C6-C7-C8-C9	C7-C8-C9-O12	H-C7-C8-H	>C7-C8-C9	C6-C7	C7-C8	C8-C9	C9-O12
CI_{PYR}	-179.5	104.9	-11.0	65.6	101.5	1.41	1.45	1.38	1.25
CI_{PYR*}	179.4	-105.4	11.4	-65.1	101.5	1.41	1.45	1.38	1.25
CI_Z	173.2	75.7	-1.2	97.9	111.9	1.38	1.45	1.32	1.29
CI_E	178.8	131.2	-21.8	54.7	112.1	1.41	1.43	1.42	1.22
CI_{perp}	154.0	-98.5	1.2	-83.3	114.7	1.40	1.44	1.30	1.32
$CI_{Ethylidene}$	180.0	180.0	0.0	-	138.3	1.52	1.48	1.43	1.20
CI_{HT}	178.8	-48.5	95.5	-108.6	130.3	1.46	1.39	1.44	1.21

Table S11. Energies of the conical intersections (absolute values in Hartree and energy differences in eV) optimized at the SA2-CASSCF(6,6). Energies calculated using XMS-CASPT2(6,6) and XMS-CASPT2(12,11). The reference value is the lowest energy conical intersection. We present the energies computed with larger active space only for the most stable CIs.

	**Absolute energy (in a.u)**		**ΔE (in eV)**		**Absolute energy (in a.u)**		**ΔE (in eV)**	
**CI**	**CASSCF (6,6)**	**XMS (6,6)**	**CASSCF (6,6)**	**XMS (6,6)**	**CASSCF (12,11)**	**XMS (12,11)**	**CASSCF (12,11)**	**XMS (12,11)**
CI_{PYR}	-875.79248	-878.29450	0.00	0.00	-875.83915	-878.28568	0.00	0.00
CI_{PYR*}	-875.79248	-878.29446	0.00	0.00	-875.83856	-878.28566	0.02	0.00
CI_{Z}	-875.77725	-878.28696	0.41	0.20	-875.80744	-878.27723	0.85	0.23
CI_{E}	-875.78065	-878.28244	0.32	0.33	-875.82685	-878.27587	0.33	0.27
CI_{perp}	-875.75291	-878.27074	1.08	0.65				
$CI_{Ethylidene}$	-875.77523	-878.24273	0.47	1.41				
CI_{HT}	-875.75223	-878.26001	1.10	0.94				

Note S11: Linear interpolations in internal coordinates (LIICs)

The potential energy curves connecting the selected conical intersections and their respective S_1 minimum are shown in supplementary information Fig. S15 and Fig. S16. The geometries of the minimum and conical intersections were optimized using SA-CASSCF(6,6)/6-31G(d). The energies along the LIIC were computed at XMS-CASPT2(12,11)/6-31G(d).

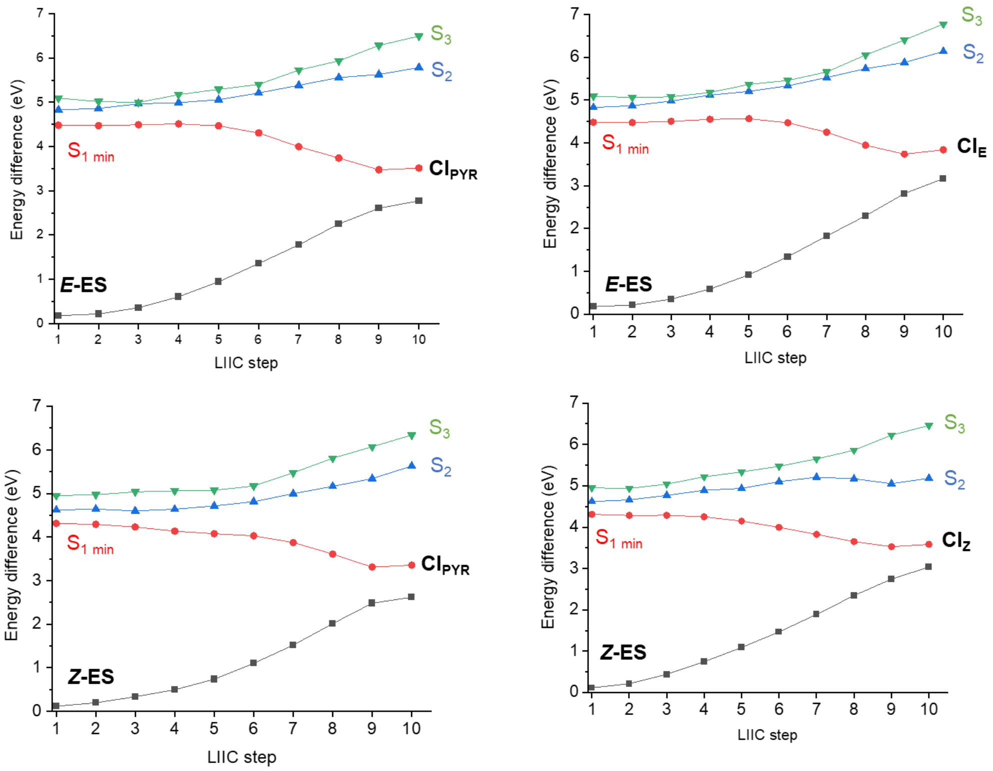

Fig. S15. Potential energy curves computed from the S_1 minimum to CI at XMS5-CASPT2(12,11)//SA-CASSCF(6,6).

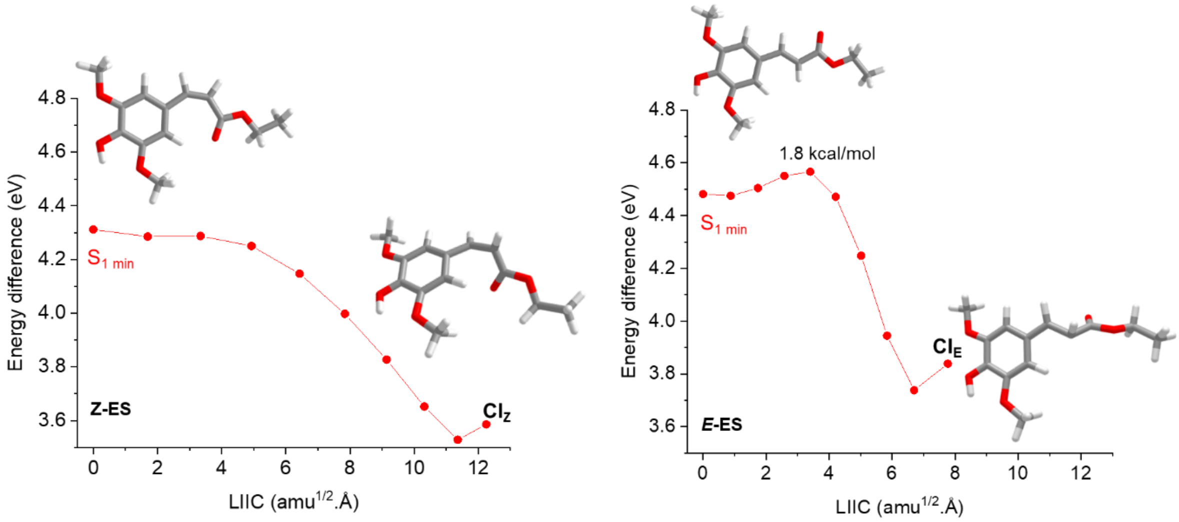

Fig. S16. Potential energy curves computed from the S_1 minimum to CI at XMS5-CASPT2(12,11)//SA-CASSCF(6,6) showing only the S_1 state to emphasize the energy barrier.

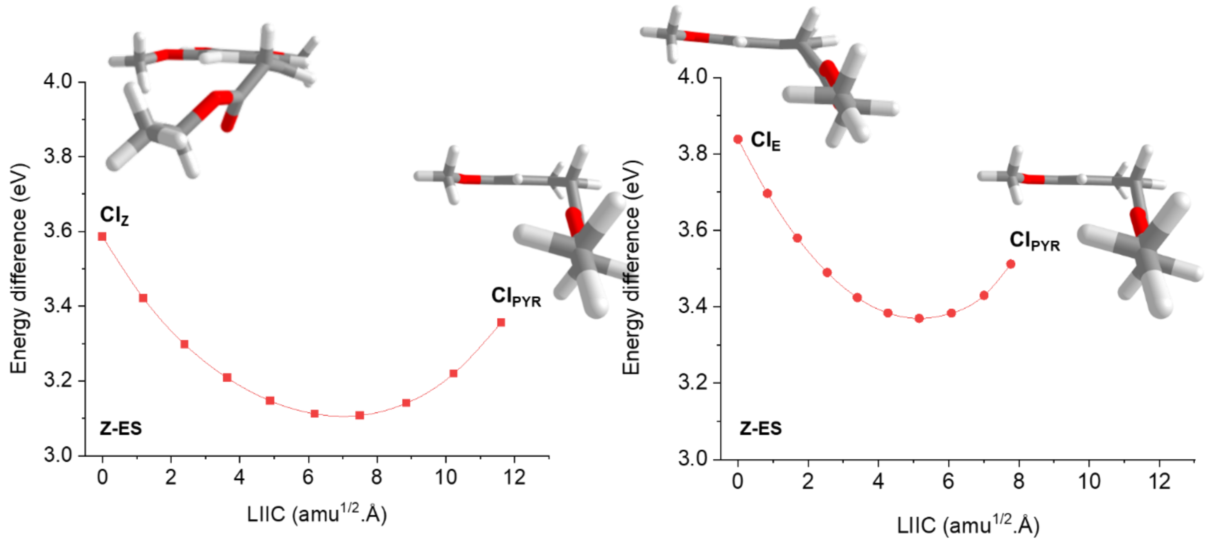

Fig. S17. Potential energy curves computed from CI_Z to CI_{PYR} (left) and from CI_E to CI_{PYR} (right) at XMS5-CASPT2(12,11)//SA-CASSCF(6,6).

Note S12: Topography of the conical intersections

It is well known that the topography of CIs can govern the dynamics of a system and, consequently, the resulting photoproduct yield.[22-24] To understand the factors which control photoisomerisation in ES, we looked at the topography of the branching plane (BP), i.e. the shape of the potential energy surface close to the CIs. The branching plane is controlled by two molecular modes: the electronic gradient difference ($\vec{g}$) and the nonadiabatic coupling (NAC) vector ($\vec{h}$) between two states. The last one measures the mixing between the adiabatic electronic states and the nuclear motions. These modes indicate possible photochemical processes mediated by a particular conical intersection.

In the vicinity of CIs, the probability for the population to return to the upper state depends on the inclination and symmetry of the double cone, which defines the intersection.[24] The full characterization of a CI is given by the parameters Δ_{gh}, σ , θ_s, and δ_{gh}. The asymmetry of the intersection is given by Δ_{gh} where, for a symmetric intersection, $\Delta_{\mathrm{gh}} = 0$, σ is the relative tilt, i.e. how much the intersection plane is tilted with respect to the xy plane. The tilt angle (θ) defines the angles between these two planes; δ_{gh} is the strength or pitch of the branching plane, given by the sum of energy difference in *x* and *y* directions.

The energy of two states in the branching space is given by the following Equation:

$$E^A(x,y), E^B(x,y), = E^X + s^{AB}x + s^{AB}y \pm \delta_{gh}\sqrt{(x^2+y^2) + \Delta_{gh}(x^2-y^2)} \quad (6)$$

Where s^{AB} is the gradient sum vector and E^X is the energy at the intersection point. The topographies of the CIs (supplementary information Fig. 18 and Fig. 19) are given by this equation, considering the average energy $E^X = 0$. The modes that drive the excited population to a conical intersection in the $\vec{g}$ direction are the bond-length alternation (BLA) stretching and the hydrogen out-of-plane (HOOP) wagging, and the torsional ϕ twisting in the $\vec{h}$ direction. Note that in the S_0 surface there is one preferential direction along $\vec{g}$ but no preferential direction along $\vec{h}$.

The surfaces described by equation (6) can be classified as (*i*) peaked or sloped, or (*ii)* single-path or bifurcating. [24] If the intersection point is a minimum of the higher surface in the branching plane, the surface is peaked; if at some point the energy of the higher state becomes lower than the intersection, it is characterized as sloped; if there is one preferential pathway for relaxation, the CI is classified as a single-path; and if there are two directions, it is bifurcating. Peaked intersections are considered to be more efficient since they reduce the possibility of "up-funneling", i.e. the population's recrossing back to the excited-state.[25] According to Galvan's classification,[24] when P > 1, the CI is classified as sloped and when B > 1 as a single path. However, it is worth noticing that this is valid only in the immediate vicinity of the intersections and the final products will be determined by the topography of the surfaces farther away from the intersections. Furthermore, this model does not take into account dynamical effects that should also play a role on the determinations of the final outcomes.[26]

The parameters related to the topography of the CIs as presented above are shown in supplementary information Table S12. Neither one of the CIs are symmetric ($\Delta_{\mathrm{gh}} > 0$). The degree of sloped character, along $\vec{g}$ is reflected in the value of σ and along $\vec{h}$ is reflected in θ_s. These parameters suggest that slope is much higher in the $\vec{g}$ and smaller in the $\vec{h}$. The different

parameters computed for the intersections indicate that CI_E, CI_Z and CI_{PYR} have different behavior and, therefore, different reactivity.

Table S12. Topography parameters for the S_1/S_0 conical intersections (in atomic units and degrees). When P > 1, the conical is classified as sloped and when B > 1, as a single path.

**Structure**	**P**	**B**	**δgh (H/au)**	$\boldsymbol{\Delta_{gh}}$	**σ (H/au)**	$\boldsymbol{\theta_s}$	$\mathbf{S^{AB}x}$ **(H/au)**	$\mathbf{S^{AB}y}$ **(H/au)**
CI_{PYR}	1.85	1.71	0.0688	0.5192	1.6724	0.04	0.1150	0.0049
CI_{PYR*}	1.88	1.72	0.0685	0.5121	1.6852	0.04	0.1153	0.0044
CI_Z	8.26	2.85	0.0691	0.4620	3.4743	0.01	0.2399	0.0032
CI_E	4.98	3.72	0.0599	0.2560	2.4873	0.12	0.1477	0.0184

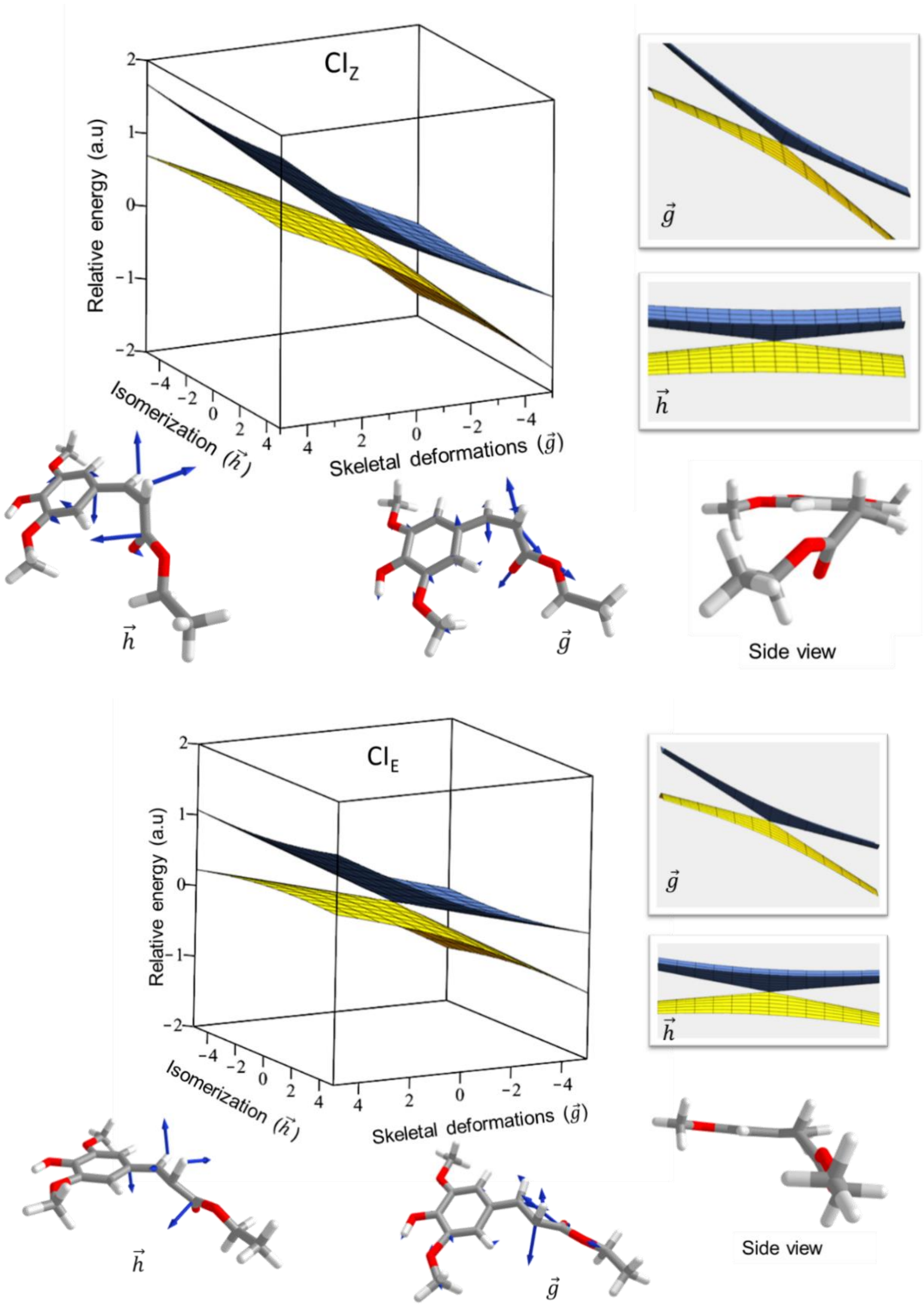

Fig. S18. Topography of the CIs around CI$_E$ and CI$_Z$. The cuts along $\vec{g}$ and $\vec{h}$ are highlighted on the right side of the Figure. The MECI is sloped in the direction of the gradient (which corresponds to the direction of approaching to the CI), but peaked in the direction of the NAC. The blue arrows in the molecules represent the direction of the displacement modes, which mainly contribute to the gradient difference vector and to the nonadiabatic coupling vector. The gradient difference corresponds to skeletal deformations mainly involving bond-length alternation; for CI$_{E,}$ it also involves the HOOP mode. The $\vec{h}$ is dominated by the torsional motion leading to planarization.

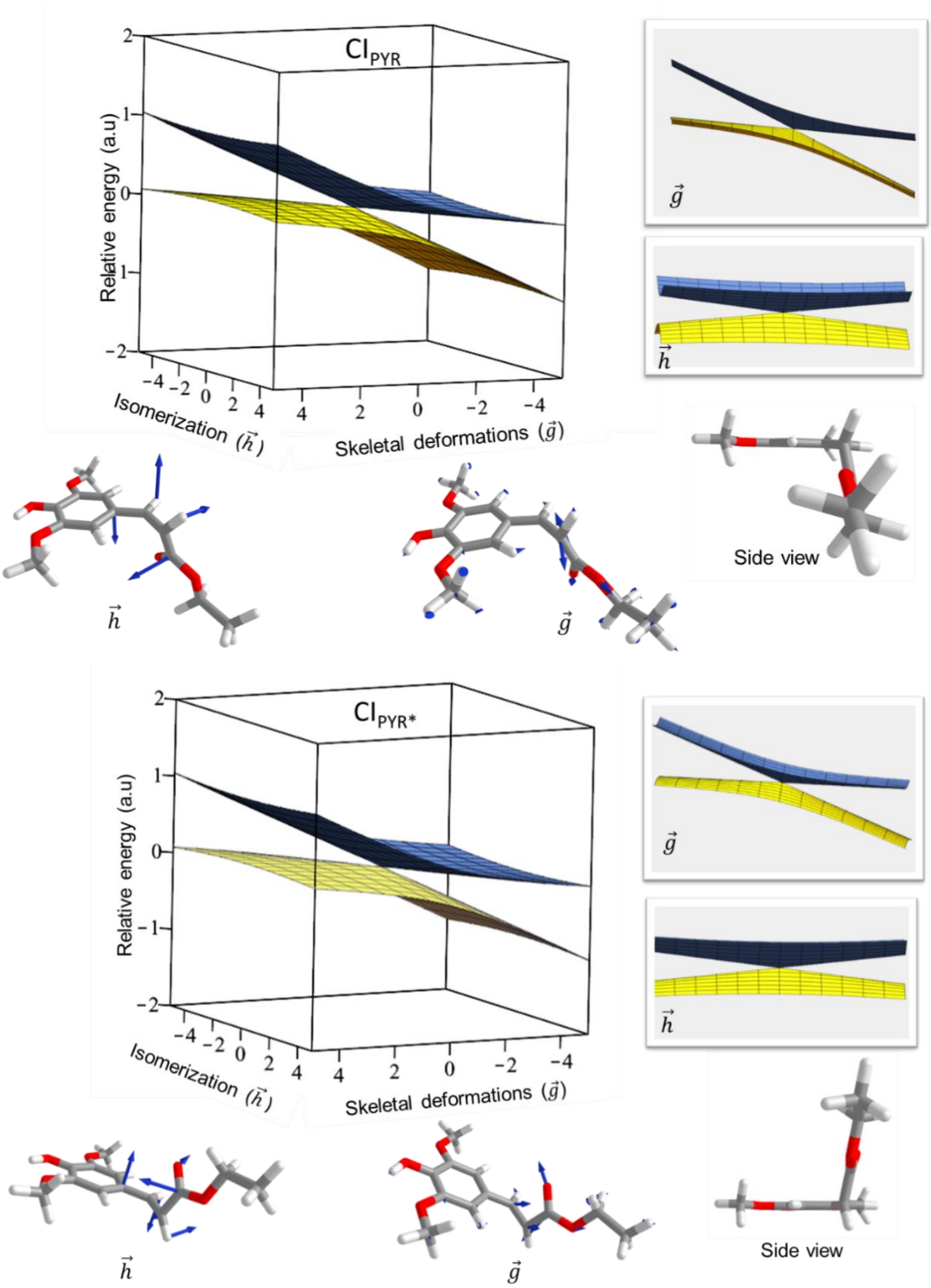

Fig. S19. Topography of the CY$_{PYR}$ and CY$_{PYR*}$ CIs. They have equivalent energy but are rotated to opposite directions (clockwise and anticlockwise). The cuts along $\vec{g}$ and $\vec{h}$ are highlighted on the right side of the Figure. The MECI is sloped in the direction of the gradient (which corresponds to the direction of approaching the CI), but peaked in the direction of the NAC. The blue arrows in the molecules represent the direction of the displacement modes which mainly contribute to the gradient difference vector and to the nonadiabatic coupling vector. The gradient difference corresponds to skeletal deformations mainly involving bond-length alternation and HOOP mode. The $\vec{h}$ is dominated by the torsional motion leading to planarization.

Supplementary References

1. Turner, M. A. P., Turner, R. J., Horbury, M. D., Hine, N. D. M. & Stavros, V. G. Examining solvent effects on the ultrafast dynamics of catechol. *J. Chem. Phys.* **151**, 084305 (2019).
2. Whittock, A. L. *et al.* Reinvestigating the Photoprotection Properties of a Mycosporine Amino Acid Motif. *Front. Chem.* **8**, 574038 (2020).
3. Abiola, T. T. *et al.* New Generation UV-A Filters: Understanding their Photodynamics on a Human Skin Mimic. *J. Phys. Chem. Lett.* **12**, 337-344 (2021).
4. Horbury, M. D., Flourat, A. L., Greenough, S. E., Allais, F. & Stavros, V. G. Investigating isomer specific photoprotection in a model plant sunscreen. *Chem. Commun.* **54**, 936-939 (2018).
5. Hanson, K. M., Narayanan, S., Nichols, V. M. & Bardeen, C. J. Photochemical degradation of the UV filter octyl methoxycinnamate in solution and in aggregates. *Photochem. Photobiol. Sci.* **14**, 1607-1616 (2015).
6. Hanson, K. M., Narayanan, S., Nichols, V. M. & Bardeen, C. J. Correction: Photochemical degradation of the UV filter octyl methoxycinnamate in solution and in aggregates. *Photochem. Photobiol. Sci.* **15**, 1212-1212 (2016).
7. Grubb, M. P., Orr-Ewing, A. J. & Ashfold, M. N. KOALA: A program for the processing and decomposition of transient spectra. *Rev. Sci. Instrum.* **85**, 064104 (2014).
8. Abiola, T. T. *et al.* Towards developing novel and sustainable molecular light-to-heat converters. *Chem. Sci.* **12**, 15239-15252 (2021).
9. Baker, L. A. *et al.* Ultrafast photoprotecting sunscreens in natural plants. *J. Phys. Chem. Lett.* **7**, 56-61 (2015).
10. Zhao, X., Luo, J., Yang, S. & Han, K. New Insight into the Photoprotection Mechanism of Plant Sunscreens: Adiabatic Relaxation Competing with Nonadiabatic Relaxation in the cis→ trans Photoisomerization of Methyl Sinapate. *J. Phys. Chem. Lett.* **10**, 4197-4202 (2019).
11. Snellenburg, J., Laptenok, S., Seger, R., Mullen, K. M. & Van Stokkum, I. H. M. Glotaran: A Java-based graphical user interface for the R package TIMP. *J. Stat. Softw.* **49**, 1-22 (2012).
12. Mullen, K. M. & Van Stokkum, I. H. M. TIMP: an R package for modeling multi-way spectroscopic measurements. *J. Stat. Softw.* **18**, 1-46 (2007).

13 Yanai, T., Tew, D. P. & Handy, N. C. A New Hybrid Exchange-Correlation Functional Using the Coulomb-Attenuating Method (CAM-B3LYP). *Chem. Phys. Lett.* **393**, 51-57, doi:DOI: 10.1016/j.cplett.2004.06.011 (2004).

14 Gaussian 16 v. Revision C.01 (Gaussian, Inc., Wallingford, CT, 2016).

15 Malmqvist, P.-Å. & Roos, B. O. The CASSCF state interaction method. *Chem. Phys. Lett.* **155**, 189-194, doi:http://dx.doi.org/10.1016/0009-2614(89)85347-3 (1989).

16 Finley, J., Malmqvist, P.-Å., Roos, B. O. & Serrano-Andrés, L. The multi-state CASPT2 method. *Chem. Phys. Lett.* **288**, 299-306, doi:http://dx.doi.org/10.1016/S0009-2614(98)00252-8 (1998).

17 Shiozaki, T., Győrffy, W., Celani, P. & Werner, H.-J. Communication: Extended multi-state complete active space second-order perturbation theory: Energy and nuclear gradients. *J. Chem. Phys.* **135**, 081106, doi:10.1063/1.3633329 (2011).

18 Ghigo, G., Roos, B. O. & Malmqvist, P.-Å. A modified definition of the zeroth-order Hamiltonian in multiconfigurational perturbation theory (CASPT2). *Chem. Phys. Lett.* **396**, 142-149, doi:http://dx.doi.org/10.1016/j.cplett.2004.08.032 (2004).

19 Forsberg, N. & Malmqvist, P.-Å. Multiconfiguration perturbation theory with imaginary level shift. *Chem. Phys. Lett.* **274**, 196-204 (1997).

20 Fdez. Galván, I. *et al.* OpenMolcas: From Source Code to Insight. *J. Chem. Theory Comput.* **15**, 5925-5964, doi:10.1021/acs.jctc.9b00532 (2019).

21 Ko, C. *et al.* Ab Initio Excited-State Dynamics of the Photoactive Yellow Protein Chromophore. *J. Am. Chem. Soc.* **125**, 12710-12711, doi:10.1021/ja0365025 (2003).

22 Yarkony, D. R. On the adiabatic to diabatic states transformation near intersections of conical intersections. *J. Chem. Phys.* **112**, 2111-2120, doi:10.1063/1.480779 (2000).

23 Yarkony, D. R. Nuclear dynamics near conical intersections in the adiabatic representation: I. The effects of local topography on interstate transitions. *J. Chem. Phys.* **114**, 2601-2613, doi:10.1063/1.1329644 (2001).

24 Fdez. Galván, I., Delcey, M. G., Pedersen, T. B., Aquilante, F. & Lindh, R. Analytical State-Average Complete-Active-Space Self-Consistent Field Nonadiabatic Coupling Vectors: Implementation with Density-Fitted Two-Electron Integrals and Application to Conical Intersections. *J. Chem. Theory Comput.* **12**, 3636-3653, doi:10.1021/acs.jctc.6b00384 (2016).

25 Ben-Nun, M., Molnar, F., Schulten, K. & Martínez, T. J. The role of intersection topography in bond selectivity of cis-trans photoisomerization. *Proceedings of the National Academy of Sciences* **99**, 1769, doi:10.1073/pnas.032658099 (2002).

26 Sellner, B., Barbatti, M. & Lischka, H. Dynamics starting at a conical intersection: Application to the photochemistry of pyrrole. *J. Chem. Phys.* **131**, 024312, doi:10.1063/1.3175799 (2009).
